# Training competent and effective Primary Health Care Workers to fill a void in the outer islands health service delivery of the Marshall Islands of Micronesia

**DOI:** 10.1186/1478-4491-4-27

**Published:** 2006-12-15

**Authors:** Bhalachandra H Keni

**Affiliations:** 1 Outer Islands Health Care System, Ministry of Health, PO Box 16, Majuro, Republic of Marshall Islands

## Abstract

**Background:**

Human resources for health are non-existent in many parts of the world and the outer islands of Marshall Islands in Micronesia are prime examples. While the more populated islands with hospital facilities are often successful in recruiting qualified health professionals from overseas, the outer islands generally have very limited health resources, and are thus less successful. In an attempt to provide reasonable health services to these islands, indigenous people were trained as Health Assistants (HA) to service their local communities. In an effort to remedy the effectiveness of health care delivery to these islands, a program to train mid-level health care workers (Hospital Assistants) was developed and implemented by the Ministry of Health in conjunction with the hospital in Majuro, the capital city of the Marshall Islands.

**Methods:**

A physician instructor with experience and expertise in primary health care in these regions conducted the program. The curriculum included training in basic health science, essentials of endemic disorders and their clinical management appropriate to the outer islands. Emphasis was given to prevention and health promotion as well as to the curative aspects. For clinical observation, the candidates were assigned to clinical departments of the Majuro hospital for 1 year during their training, as assistants to the nursing staff. This paper discusses the details of the training, the modalities used to groom the candidates, and an assessment of the ultimate effectiveness of the program.

**Results:**

Out of 16 boys who began training, 14 candidates were successful in completing the program. In 1998 a similar program was conducted exclusively for women under the auspices of Asian Development Bank funding, hence women were not part of this program.

**Conclusion:**

For developing countries of the Pacific, appropriately trained human resources are an essential component of economic progress, and the health workforce is an important part of human resources for sustainable progress and development.

## 1 Background

The Outer Islands Health Care System (OIHCS) is an integral component of the Bureau of Primary Health Care (PHC), in the Republic of Marshall Island's (RMI) Ministry of Health (MOH). The OIHCS is responsible for the operations of the primary health care services to approximately 17 000 people dwelling on 19 atolls in an area of 1 812 992 sq. km. of Pacific Ocean. Outer island health care needs are met through a referral network of 52-health centers each of which is operated by a "Health Assistant". The Health Assistants are mid level practitioners, who single-handedly run the health center. Physician assistance is available to provide guidance as needed by 2-way radio [1]. During the fiscal year 2002 (Oct 2001-Sep 2002), the primary health care network registered 30 481 visits in the various health centers in the outer islands. Seventy seven percent (77%) or 23 740 of those visits were for curative services. Prenatal and postnatal care represented 1.9% (594) and 0.36% (110) respectively. The amount of visits recorded in child health clinics represented 1.7% (533) and family planning/reproductive health services, 3.3% (1 004). Patients with dental disorders comprised 11.5% (3 531) of the visits.

A total of 182 live births were registered in the outer islands. Three stillbirths were recorded. There were 22 infant deaths recorded.

Growing staff shortages led to the closure of several health centers, seriously impacting the health service to the community. Provision of sufficient numbers of properly trained health care workers to deliver adequate health services became a matter of urgency.

The focus of this paper is a description of one attempt to fill the void of medical care in a community where there is no doctor. It examines the effectiveness of training indigenous people to become "Health Assistants", whose responsibility is to serve the community in a wide range of routine and emergency medical situations.

The Republic of the Marshall Islands (RMI) is situated in the Central Pacific Ocean between 4°N and 14°N and 160°E and 173°E in two parallel chains of 31 atolls and islands. The Eastern Ratak (Sunrise) chain consists of 15 atolls and islands, and the Western Ralik (Sunset) chain of 16 atolls and islands. The total number of islands and islets is about 1 225. RMI with 24 inhabited atolls has a population of over 50 000. Each atoll consists of a ring of islets encircling a deep-water lagoon. The islets are interconnected and surrounded by a coral reef. None of these low-lying land areas have an elevation greater than ten feet above sea level.

The Ministry of Health (MOH) with its health care network serves the needs of the populace through its primary, secondary and tertiary components (see Table 1).

**Table 1:** Divisions of PHC and populations densities

**Division**	**No of atolls**	**Population**
1. Majuro	1	23 676
2. OIHCS	18	14 170
3. Ebeye	1	10 902
4. Program 177	4	2 092

**Total**	**24**	**50 840**

*1999 Census

Health Assistants (HA) are front-line health workers in the community who have been trained to carry out primary health care services[2]. Traditional birth attendants still play a major role in conducting the delivery and postnatal care, in most parts of the outer islands and especially where there are only male health workers. However, Health Assistants do assist as needed. The Health Assistants are available around the clock in the remote outer islands, where normal living comforts are virtually non-existent. Health Assistants collaborate with the Community Health Councils to promote and foster the concept of a shared responsibility for health.

## 2 Resources & methods

### Structure & facilities

The Ministry of Health has a well-established infrastructure for the health sector. The department of OIHCS has a permanent teaching and training block to house 30 – 50 candidates at a time.

A brightly illuminated classroom measuring about 1.2 × 3.6 metres with two lengthy black boards attached to two adjacent walls provides excellent visibility and an atmosphere conducive to learning. The room includes state-of-the-art audiovisual equipment and educational materials such as posters, charts, clay models, and mannequins. The classroom is well equipped with desks, chairs, cupboards, writing materials and other appropriate furniture.

### Selection criteria

Various channels of advertising media were used to spread the message in the outer islands to recruit prospective applicants. Applications were restricted to residents of the outer islands who had successfully completed high school graduation. Each candidate had to be either nominated by the respective local mayor or health council of the atoll. The candidates were subjected to a competitive written test. The committee selected 16 candidates who scored above an acceptable grade level in the pre-test of 64 applicants. All 16 youths were unemployed and had an average age of 26 years at the time of application.

The training program demanded a high degree of commitment and effort from the participants in order that they might become responsible health service providers. As an entire community depends on them for their health needs in situations of life and death, it becomes imperative that they are molded into disciplined Health Assistants with high standard of ethics and morals. Hence, strict rules and regulations were instituted, including punctuality, a dress code, and compulsory classroom cleaning and maintenance exercises.

The "Health Assistants Training Program" (HATP) began with an orientation, and an overview of the scope and purpose of the health assistant program.

### Classroom environment & teaching processes

The classroom atmosphere was comfortable but warm. Initially, for the first 5 months of the program, students attended classes for 8 hours a day with periodic breaks. After 5 months, the hospital assignments began in the morning session (from 8:00 to 12:00), and the theory lectures were held in the afternoon session (from 13:00 to 17:00). Small-group work case studies, brainstorming, role-playing, presentations and classroom lecture methods were used. There was close contact between the trainer and the trainees. Students were given full freedom to express their views.

#### 2.1.1 English & new medical terms

Lack of familiarity with the English language was one of the major barriers amongst the students of the Marshall Islands. In an effort to overcome the language barrier and to familiarize students with medical terminology, one black board was exclusively provided so that a vocabulary of 'new words' could be recorded. These words remained on the board for several months with the expectation that repeated exposure would facilitate memorization. It is the author's opinion that daily sighting and noticing of these words has some positive impact on the retention of these words in the student's memory bank. It is the reverse of the proverb 'Out of sight is out of mind'. English terms, particularly medical jargon, were explained using practical examples until the trainees understood and could translate the new words into their own mother tongue, Marshallese.

#### 2.1.2 Mathematics

Basic mathematics had to be reviewed and reinforced so that students could accurately calculate percentages, pharmacological dosages of medications and other problems requiring mathematical skills. Regular practices of mathematics skills were challenged on an ongoing basis.

#### 2.1.3 Health science and basic anatomy & physiology

The health science training began with the art and science of 'living beings', followed by a more intensive exploration of the anatomy and physiology of the human body. The absence of cadaver dissection was a disadvantage in understanding the depth of anatomical configurations. However, with the aide of audio-visual *Delmar's Anatomy and Physiology *transparencies, clay models and charts, the essentials of human anatomy were successfully taught.

The fundamentals of pharmacology were also addressed, and the method and mechanisms involved in 'inventory system and management' in the outer islands were highlighted. For practical demonstration purposes the students were periodically involved in receiving, sorting, placing and dispatching the medication to outer islands health centers from the OIHCS formulary. Step by step, the entire formulary in the OIHCS pharmacy store was theoretically covered.

Theory also included basics of pathology, ophthalmology, ENT, surgery and medicine applicable to conditions in the Marshall Islands. Essentials of community medicine and public health were addressed in detail. Both theory and practical knowledge were emphasized in obstetrics, and a compulsory practice in conducting deliveries was instituted. Management of serious illnesses in children was repeatedly covered, including the practice of neonatal infusions.

#### 2.1.4 The hospital assignments

After 5 months of rigorous theory teaching, students were given hospital assignments as an introduction to the hospital-working environment and patient care. Practical hospital rotations went from 8:00 to 12:00, five days a week. Students were circulated into various wards including surgery, medicine, maternity, pediatrics, the out patient department (OPD), emergency room (ER), operating room (OR) and the public health department under the supervision of the Chief Nurse. Students were also exposed to the medical laboratory and the dental department.

#### 2.1.5 Bedside clinical training

There is much that can be gained from teaching at the bedside. It allows trainees to learn professionalism and to grasp the principles of communication with real patients [3]. Each student had to take detailed histories and perform physical examinations of the assigned patient using standard medical school technique, and had to present the clinical case to the entire class and the trainer. The emphasis was not on the diagnosis, but rather the ability of the student to make a sound decision regarding how critical the case may be, and whether or not the patient should be treated at the health center or to transfer to the referral hospital either at Majuro or Ebeye. However, the technique of differential diagnosis, the process of elimination, and how to arrive at a reasonable conclusion regarding the affected systems were all taught. The practical application of theoretical knowledge was employed by the students in history taking, general examination, assessment and plan of action and management of the hospital inpatients from the point of view of health practitioners from the outer islands.

#### 2.1.6 Teamwork & organized membership

In addition to classroom teaching and practical demonstrations, 'group discussions and competitive sessions' were organized in the form science quizzes and debates, in an effort to inculcate a team spirit. Cooperative learning is based on positive interdependence and the idea that group members need each other to complete the group's work. Without structure it is common that one person ends up doing most of the work or dominating the group, while others end up with minimal participation [4].

#### 2.1.7 Communication skills for health promotion

Special emphasis was given to communication skills and personality development. In the final hour of the day, a session was devoted exclusively to the art of communication and presentation. Each candidate was asked to make a verbal presentation in English using a specific format. Wide ranges of health promotion and prevention topics were provided, although students were encouraged to select a topic independently. The format was designed to guide students through the necessary elements of conducting research on any given topic.

At the end of verbal presentation, each fellow trainee was encouraged to ask questions or challenge the presenter in some way. In addition each student was expected to contribute one point to a discussion regarding what had been learned from the presentation. The author has found this to be a valuable technique to improve the "listening ability, concentration, attentiveness, and comprehension" of the students. This practice also helped to reduce shyness, stage fright, and allowed for greater freedom of expression, particularly as the trainees faced guest lectures by foreigners.

## 3 Results

The candidates were subjected to closed book examinations at suitable intervals, completing 3 tests and one final examination, in addition to several small surprise tests and quizzes.

**Figure 1:**
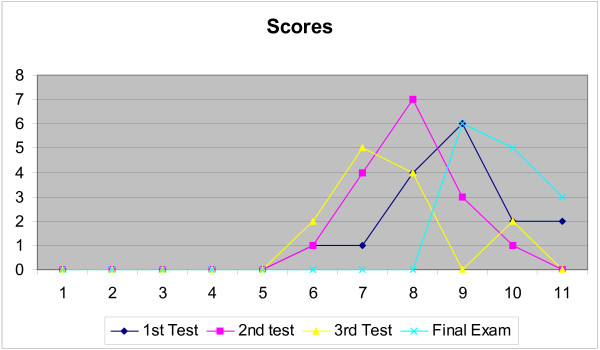
Results of the periodical assessments conducted during the year 2003–2004.

As shown in Figure 1, the scores of the 2nd test showed a normal distribution pattern with mean, median and mode at 64. The scores for the 3rd test had a bimodal distribution. Final exam scores were positively skewed with Median of 82.

Improvement in terms of enhanced services (see Table 2):

• Eight new health centers came into operations

• New graduates replaced four retired Health Assistants.

**Table 2:** Number of health centers in operation after the new graduates' posting

Health Centers	2004	2005
Closed	14	6
Operating	44	52

Total	58	58

## 4 Evaluation

In the final month the trainees were evaluated independently, and found to possess satisfactory knowledge and curative skills by both the Hospital Internist and Staff Surgeon.

By the end of the course all the students showed good communication skills and confidence in verbal presentation, which were demonstrated in a field trip organized by OIHCS. The students ventured out to a neighboring atoll, where they conducted a meeting with the local heads of the government, and organized health talks in the community and at the school. The students also conducted school health screening for anemia and oral health. During the day of the field trip, the students managed the health center activities. They examined all the out patients and prescribed and distributed the appropriate medications. However, the trainer, who intervened only when absolutely necessary, supervised all activities.

## 5 Discussion

### Historical background

China faced a medical crisis in 1949 when health conditions were abysmal, the majority of people suffered from poor health, and there was almost no access to modern medicine. During that Cultural Revolution, medical schools ceased their routine instructive functions and no new students were admitted between 1966 and 1970. Instead, more emphasis was placed on part-time health workers such as the 'barefoot doctors (*chizu yisheng*) in the countryside and **'red medical workers' **(*hongyi*) in the urban neighborhoods. It was known that barefoot doctors contributed the bulk of basic medical care in the rural areas during that era [5].

The World Health Organization acknowledged China's achievements, for their mobilization of part-time workers and bringing significant improvement in the overall health of the community and offering affordable health care to the rural population. The World Bank cited China's reliance on low levels of technology and community services as a model for other developing nations [6].

In the mid-1970s the Northern Territory Department of Health and Community Services, Australia developed an Aboriginal Health Worker (AHW) training system, which was based on the recognition of inseparable components of health education and provision of Primary Health Care services. In 1979 the House of Representatives Standing Committee on Aboriginal Affairs reported that AHW training served as a model upon which the state training programs were based.

In the mid-1940s in the Pacific, military corpsmen in the Marshall Islands were trained on the job for one year to fill the post-war shortage of health care providers. Then in the 1970s this corpsmen were trained to be mid level practitioners – 'Health Assistants'. The corpsmen trained around 80 Health Assistants over 30 years. The health workers in Marshall Islands who graduated from this program have played a key role in health care delivery. The gradual decrease in the number of the Health Assistants was due to the natural demographic changes, retirement and death. Social and economic factors have also contributed to the migration and career changes of these Health Assistants. At the end of 2003, there were 49 Health Assistants remaining on the staff role.

The World Health Organization (WHO) has been sustaining this training concept as an alternative arrangement for meeting the needs of the growing demand for human resources for health. WHO has supported the training of the health workers (with differing job titles) in Kiribati, Cook Islands, Samoa, Vanuatu, Solomon Islands, Federated States of Micronesia and the Marshall Islands.

### Research

Research suggests that medical knowledge and techniques are continually expanding and there is a relative neglect of continuing education for all personnel working in the health sector [7]. The most powerful way to affect the health and welfare of the community is through the improved understanding of the awareness of health. The most acceptable way of communicating this message is through developing the indigenous health worker.

Recruiting trained medical doctors to remote atolls serving populations of only a few hundred is rarely possible from either a fiscal or practical perspective. These medical experts demand high salaries, and generally service much larger populations. Thus, enlisting indigenous medical assistants who have been appropriately trained to oversee the health needs of these smaller populations seems a reasonable solution [8]. This program succeeded in opening up employment opportunities and motivating hard-working young people.

The training of indigenous personnel is considered a government priority, as the lack of a well-trained indigenous workforce remains one of the main impediments to progress in health development. An inordinate proportion of the health budget is spent on the salaries of expatriate doctors, dentists and nurses due to the lack of a well-trained national health workforce [9].

### First hand experience

This training program utilized the services of a fulltime medical doctor, from the initial planning and execution stages, to the theoretical classroom teaching, clinical hospital rotations, field trips and graduation. Hence, through the 18-month program, potential problems regarding consistency and quality of information were averted [10]. The curriculum and teaching methods were designed by the trainer given the unique requirements of RMI, and were not adopted from overseas.

The trainer undertook a series of field visits to 9 atolls and 30 Health-Centers and conducted household surveys in order to assess the health status of the community prior to beginning the training program. The trainer spent 1–2 weeks in each atoll residing at the health center, as commercial provision for lodging is unavailable. The first hand experience observing medical assistants in action in various atolls proved an invaluable asset in designing the curriculum. Careful note was taken of the wide range of challenges faced by the medical assistants so that these could be incorporated into the training program. This ensured that the training was both appropriate and relevant to the areas to be serviced. Each routine task carried out by Health Assistants was're-enacted' in the classroom, so as to prepare the trainees for their future careers.

In the past, the training of Health Assistants in Micronesia was focused on the promotion of community health issues. It was very basic, and woefully inadequate for the advanced diagnostic reasoning required for clinical curative care [11]. Hence, the introduction of a program that incorporated clinical training was a significant step towards enhancing the quality of care provided on outer island atolls. Preparing medical assistants to make assertive treatment decisions, particularly regarding referrals to larger centers, is a skill that is both necessary and critical to quality patient care.

It has been argued that a holistic approach to health services requires that health workers be trained to function as clinicians and managers. A clinician without managerial training will have difficulty administering health services at various levels [12].

Administrative skills are commonly overlooked in the training of Health Assistants, as the course instructors often lack formal managerial training and experience. Fortunately, in this case, the principal trainer had postgraduate business management qualifications in addition to his medical education. He also had over 10 years of practical experience with hospital administration, health care management and nurse training.

Continuing education of the community health worker (Health Assistants) is essential to their long-term effectiveness, and is an important strategy for increasing awareness of current health issues in the community [13].

Health Assistants represent one of the largest work forces (21%) in the entire Health Ministry. With collaboration and assistance from developed countries, cost effective development of human resources for health can be continued in RMI indefinitely. The Public Service Commission of RMI employs the Health Assistants soon after the successful completion of the training program. There is a growing need of these types of Health Assistants throughout Micronesia. With good quality training and supervision they can make a major contribution to the health of the nation

## 6 Conclusion

An appropriate response to the growing health needs of those populations who have limited access to health care is one which takes into account their unique health challenges in the context of their social and economic realities. The cultural acceptability of the approach targeting both community and health service provider is an important consideration. For developing countries of the Pacific, appropriately trained human resources are an essential component of economic progress [14], and the health workforce is an important part of human resources for sustainable progress and development [15]. The profile of a suitable health worker in the South Pacific must include curative capabilities as well as skills for sustainable development [16]. Sustainable development is particularly important in terms of building a strong public policy and supportive environments for health in ways which improve living conditions, support healthful lifestyles, and achieve greater equity in health, both at present and in the future [17]. Hence, training and developing the indigenous health workers is indispensable as a part of a capacity building measure in view of sustainable development.

Eventually, 14 candidates graduated with the following essential competencies to deal with the communities of the Outer Islands.

The skills developed included:

• Patient receiving, and interviewing (detailed history and assessment)

• Patient physical examinations

• Patient case management (treatment & referrals)

• Patient case documentation and reporting

• Patient case referrals to Majuro/Ebeye

• Health information and education

• Community health evaluation and monitoring

• Community health education and health promotion

• Outreach/organizing preventive health programs such as immunization and family planning

## 7 Recommendations

### Sustainability through continuing education

It is not easy for Health Assistants in remote areas to keep up their skills, but these Health Assistants are the personnel who need it most. It is difficult to arrange continuing education for those working far from Majuro, because of logistics and financial constraints. Continuing education is vital for maintaining motivation and ensuring competent practice.

A number of strategies can be effective in achieving this task. However, it is the opinion of the author that among the most effective measures is a teaching visit by a physician consultant of OIHCS for 1–2 weeks for each health center at 6-monthly intervals. Another strategy is to conduct retraining programs periodically at OIHCS/Majuro hospital, where they can come for refresher courses and practical intervention training with patients under the supervision of medical specialists. For such rotations to be meaningful, attention must be given to scheduling which is consistent with the specific clinical learning needs of the Health Assistants and which reinforce diagnostic reasoning and safe, evidence-based clinical decision-making. The refresher training would prove highly effective in light of the fact that it would be under the overall supervision of one doctor (Physician Consultant OIHCS), and the trainees would be guided appropriately throughout the course, as the doctor would be allocated full time to this cause.
